# What influences Bangladeshi Boro rice farmers’ adoption decisions of recommended fertilizer doses: A case study on Dinajpur district

**DOI:** 10.1371/journal.pone.0269611

**Published:** 2022-06-07

**Authors:** Faruque As Sunny, Linlin Fu, Md Sadique Rahman, Taonarufaro Tinaye Pemberai Karimanzira, Huang Zuhui

**Affiliations:** 1 School of Management, Zhejiang University, Hangzhou, Zhejiang, China; 2 Institute of Rural Development, Zhejiang Academy of Agricultural Sciences, Hangzhou, Zhejiang, China; 3 Department of Management and Finance, Sher-e-Bangla Agricultural University, Dhaka, Bangladesh; 4 School of Nontraditional Security Studies, Zhejiang University, Hangzhou, Zhejiang, China; 5 China Academy of Rural Development, Zhejiang University, Hangzhou, Zhejiang, China; ICAR-National Rice Research Institute, INDIA

## Abstract

Due to the combined effect of biotic and abiotic constraints, rising population pressure, and inelastic demand in the crop and horticulture sector, Bangladesh has had to adopt heavily subsidized and intensified fertilizer policies to enhance crop productivity, achieve and sustain self-sufficiency in food production, and food security provision. Although the initiative has played a vital role in boosting production, it has also invigorated the unbalanced amount of fertilizer application practices raising questions about maintaining biodiversity and ecosystem services while feeding the nation’s population. Further research in this area must thus be applied to monitor and improve this sector. This study attempts to understand the issue by investigating the factors influencing Boro rice farmers’ adoption decisions of recommended fertilizer doses. The study employs an ordered probit model with a sample selection approach. The investigation is based on collected data from 405 randomly selected farmers using a face-to-face interview method. The farmers were classified into low, middle, high and non-adopter groups. The study revealed that farmers’ age, land typology, soil water retention, knowledge, and availability of cow dung significantly influenced farmers’ decision to apply fertilizers. However, farmers’ carry an aversion to following recommendations for fertilizer application due to their ambiguity about the whole system, their current fertilizer application-seeking behavior, and the lack of understanding of the environmental benefits of adoption. These issues urge policy interventions to initiate village-based demonstration programs that synthesize better synergies between recommended dose adoption, yield amelioration, sustainable soil care, and economics.

## 1. Introduction

With population growth projected to be 27.74 percent by 2050 [1] and a need to increase food production by up to 62 percent [2], sound agricultural practices will undoubtedly be the pivot holding global security and economics in a sustainable balance. However, agricultural land shrinkage has been an issue [3] that, when amalgamated with the pressure to increase crop productivity, has invigorated many South Asian countries to adopt heavily subsidized and intensified fertilizer policies and cropland management techniques [4]. Theoretically, when farmers lack adequate fertilizer management knowledge, the production process suffers from unbalanced, inefficacious quantities of inorganic fertilizer application, thus posing a significant threat to future agriculture output [4–7].

Agriculture, still regarded as the most crucial sector for developing countries like Bangladesh, accounts for more than 38 percent of the country’s labor force [8]. Around 70 percent of the overall population is directly or implicitly reliant on agriculture [9]. The country is naturally gifted with the soil and climate favorable for growing tropical and temperate crops. Rice (*Oryza sativa*), the country’s staple food, accounts for approximately 75 percent of the national harvested area, contributing roughly 95 percent of the total national grain [10, 11]. However, the higher aggregate demand for food due to the rising population pressure [12], the annual 1 percent agricultural land shrinkage issue because of the shifting rate of agricultural land to non-agricultural uses, and the inelastic demand for crops and horticulture sectors raised the question of the country’s ability to achieve and sustainably maintain self-sufficiency in food and agro-based livelihoods. Likewise, and more to the root of nature-based factors; biotic (i.e., sheath blight, sheath rot, stem borer, and leaf roller) and abiotic (i.e., floods, drought, changes in the precipitation pattern, and variations in temperature and humidity) conditions hamper crop productivity [13–15]. Hence, to reduce the input price to maintain lower volatility in agricultural commodity prices and encourage agricultural productivity to meet the increasing demand for food, the government has adopted a fertilizer subsidy policy that aims at the overall welfare of the producers and consumers [4]. Consequently, the fertilizer consumption per hectare of arable land increased from 255.76 kg/hectare to 600 kg/hectare between 2011 and 2021 [16, 17].

In the system surrounding rice production, apart from other normative factors (i.e., environment, soil condition, irrigation, and technologies), fertilizer plays a vital role in replenishing soil nutrient stocks that crops absorb during their life cycle [18]. Although it has been reported that, in the past, fertilizer subsidies significantly contributed to yield amelioration, some recent studies suggest that their contribution to productivity escalation and overall agricultural growth has failed to show long-term improvements, particularly in the overall national economy [4]. Data between FY 2009–10 and FY 2019–20 shows Bangladesh has increased its rice production from 32 million metric tons (MT) to around 36.6 MT [19]. However, the contribution of this sector to total gross domestic product (GDP) declined from 17 to 12.65 percent [20], and the average growth of the sector hovered around 3.7 percent [21]. This could be due to the rise of more valuable substitute crop products or maybe because some implements for production do not respond well to environmental mindedness, but it more likely seems due to long-term process inefficiencies within the sector. Most farmers’ lack of scientific farming knowledge and reliance on inorganic fertilizers, compounded with inconsequential micronutrient uses and meager adoption of soil testing-based fertilizer recommendations, have deteriorated the soil substances in the country [17, 22–27]. This has driven the government to emphasize adopting sustainable intensification methods to increase yields by adjusting fertilizer use whilst decreasing disparagement to the environment [9, 28]. However, achieving change requires a relentless commitment to include people and their thoughts in the process, as most change efforts fail due to a lack of thought about the dynamics of human motivation.

According to our knowledge, previous studies have mainly focused on technical efficiency, fertilizer management techniques, fertilizer application comparisons, fertilization strategies for higher yields, and the effects of fertilizer broadcasting [6, 29–32]. Therefore, considering the above contextualization, this study first attempts to unravel factors that have influenced the farmers to adopt the Bangladesh Rice Research Institute’s (BRRI) recommended fertilizer doses for BRRI-29 rice. Understanding these factors will play an essential role in promoting balanced nutrient application. Secondly, this study attempts to shed light on farmers’ sentiments towards adoption and non-adoption to assess their sensitivity to these facilities. Finally, this study seeks to provide some suggestions to develop a more realistic fertilizer management approach that would result in environmental amelioration, efficient use of the extension resources, and enhance the sustainable agriculture practices of farmers.

## 2. Materials and methods

### 2.1 Study area, sampling procedure, and data source

The study was conducted in the northern part of Bangladesh from February to April 2021. We purposely selected the Dinajpur district, as it is the largest district among all sixteen districts situated in northern Bangladesh. Dinajpur’s economy is primarily agricultural-based, with rice being its most well-known product [33]. The majority of land in this district is medium highlands (37 percent), followed by highland (5 percent), and medium lowlands (5 percent) [27]. Rice farmers prefer to grow paddy in either medium-high or medium lowlands due to several cost-effectiveness factors. Moreover, food insecurity and poverty rates are high in these regions [34], which may allow us to observe the real-time effects of agricultural decision-making dynamics. Finally, the Boro season (December to June) was chosen as a data collection period as it is when rice production is at its peak [19], and BRRI-29 was chosen for analysis as this variety is dominant among all Boro varieties in the study area.

For this study, multistage sampling techniques were employed. Out of 13 total upazilas (sub-districts) in the Dinajpur district, three were selected using the simple random sampling technique. The optimal sample size was determined using Krejcie and Morgan’s [35] formula. A sample of at least 384 farmers was determined based on the population of 2,990,128. For an eqaual proportion of samples, we surveyed 135 farmers from each sub-district. Thus, the final sample size for the study stood at 405.

A face-to-face interview with each respondent was conducted to collect the required data. The questionnaire was translated into the local language and was pretested before finalization. The interviewed respondents were ‘BRRI rice 29’ producing farmers. The questionnaire included questions to obtain farmers’ demographic and socioeconomic conditions; institutional services and infrastructure; agro-ecology; adoption or non-adoption behavior; fertilizer knowledge; actual fertilizer use amount; environmental consciousness; basic balanced nutrient knowledge; and soil testing.

This study attempts to investigate two different decisions: factors influencing farmers’ fertilizer adoption or non-adoption and factors affecting adopters’ different levels of fertilizer application rate. Hence, the sample was first classified into adopters and non-adopters. The segmentation is based on the Bangladesh Rice Research Institute’s (BRRI’s) recommended fertilizer doses of nitrogen (urea), triple super phosphate (TSP), and muriate of potash (MoP) for ‘BRRI rice 29’ that range from a minimum of 336.97 kg/hectare to a maximum of 524.17 kg/hectare [36]. Farmers who applied these three fertilizers within the recommended amounts were listed as adopters, and others as non-adopters. Later, the fertilizer application range was divided into three categories, and the adopters were also divided into three groups (low, middle, and high applicators) according to their fertilizer application amount (described in the following section).

### 2.2 Analytical technique

The decision to adopt or not adopt the recommended fertilizer doses was analyzed based on the general utility maximization framework [37, 38]. Under this framework, farmers adopt a technology (for this study–BRRI fertilizer recommendation) only if the utility they gain from adoption is higher than non-adoption. Even though it is not possible to observe utility, farmers’ adoption decisions are observed through which their utility is indirectly inferred.

In investigating the factors affecting the adoption of the recommended dose of fertilizers, this study used an ordered probit model with sample selection–an extension of the Heckman selection model to account for selection bias [39]. The ordered probit with sample selection model deals with a two-stage decision process: first, the decision to adopt or not the recommended doses of fertilizer (selection equation); and second, the decision of adopters to adopt different recommended dosage levels of fertilizer (outcome equation).

As stated by De Luca and Perotti [40], the outcome equation can be expressed as:

$$yj=\sum_{h=1}^{H}v_{h}1(\kappa_{h-1}<x_{j}\beta +u_{1j}\leq \kappa_{h})$$ (1)

where *x*_*j*_ is the outcome covariates, *β* is the coefficient, and *u*_1*j*_ is a random-error term. The observed outcome values *v*_1_,…,*v*_*H*_ are integers such that *v*_1_<*v*_*m*_ for *i*<*m*. *κ*_1_,…,*κ*_*H*−1_ are real numbers such that *κ*_*i*_<*κ*_*m*_ for *i*<*m*. *κ*_0_ is taken as −∞ and *κ*_*H*_ is taken as +∞.

On the other hand, the selection equation can be expressed as:

$$s_{j}=1(z_{j}\gamma +u_{2j}>0)$$ (2)

where *s*_*j*_ = 1 when observed *y*_*j*_ and 0 otherwise, *z*_*j*_ is the covariates used for modeling the selection process, *γ* is the coefficient for the selection process, and *u*_2*j*_ is a random-error term.

(*u*_1*j*_, *u*_2*j*_) have a bivariate normal distribution with mean zero and variance matrix is $\left[\begin{array}{cc} 1 & p\\ p & 1 \end{array}\right]$

Let $a_{j}=z_{j}\gamma +{offset}_{j}^{\gamma }$ and $b_{j}=x_{j}\beta +{offset}_{j}^{\beta }$. This produces the log-likelihood,

$$\ln\;L=\sum_{j\notin S}w_{j}\;\ln\left\{\Phi (–a_{j})\right\}+\sum_{h=1}^{H}\sum_{\begin{array}{c} j\notin S\\ y_{j}=v_{h} \end{array}}w_{j}\;\ln\{\Phi_{2}(a_{j},\kappa_{h}-b_{j},-p)-\Phi_{2}(a_{j},\kappa_{h-1}-b_{j},-p)\}$$

where *S* represents the set of observations for which *y*_*j*_ is observed, Φ_2_(.) is the cumulative bivariate normal distribution function (with mean [0 0]′), Φ(.) is the standard cumulative normal, and *w*_*j*_ is an optional weight for observation *j*.

In the maximum likelihood estimation, instead of directly estimated *p*, atanh *p* is estimated:

$$artanh\;p=\frac{1}{2}\;\ln\;\left(\frac{1+p}{1-p}\right)$$

From the likelihood form, it is clear that if *p* = 0, the log-likelihood for the ordered probit sample selection model is equal to the sum of the ordered probit model for outcome *y* and the selection model. Thus we can perform a likelihood-ratio test by comparing the log-likelihood of the entire model with the sum of the log-likelihoods for the ordered probit and selection models.

The dependent variable for the selection model is binary (1 if the respondent adopts the recommended dose and 0 otherwise). While the dependent variable for the outcome model is categorized into three levels, the first group who applied fertilizer within 336.97 kg/hectare to 390 kg/hectare was categorized as the ‘lower bound’ applicator, followed by the ‘middle bound’ applicator that applied fertilizer within 391 kg/hectare to 450 kg/hectare, and the ‘higher bound’ applicators group that applied fertilizer within 451 kg/hectare to 524.17 kg/hectare.

Several interrelated factors proposed in various literatures are considered to influence farmers’ decisions to adopt agricultural innovations. For instance, farmers’ age [41, 42], education and gender [43, 44], household size [45, 46], farm size [38, 47, 48], land ownership [49], farming experience [50, 51], farm and non-farm income [22, 52–54], types of farm land [55, 56], family labor [57], livestock ownership [58], market distance [46, 59], irrigation facilities [60], information [61], cost-effectiveness [62], the role of extension services [63], friends and neighbors’ influence [64], credit availability [65], infrastructure [65], learning by doing [66], and application timing and methods [67–69] affect farmers’ decision to accept or reject a technology.

However, in most studies, deciding which influencing factors to present seems primarily an explorative research exercise. It also depends on several internal and external influences, which differ along with many facets of covarying factors [70–72]. Therefore, in the light of previous empirical research, the chosen explanatory variables and the hypothesis drawn for this particular study are presented below (Table 1). The positive and negative signs in Table 1 indicate the type of association between that particular variable and an adoption decision. For instance, we expect to be convinced that farmers’ education, income level, household size, knowledge of recommended fertilizer doses, and environmental awareness are more likely to influence their adoption decision positively. On the other hand, farm size and the topography of farmland are expected to negatively influence farmers’ adoption decisions.

**Table 1:** Description of the explanatory variables specified in the models.

Notation	Description	Variable type/ criteria	Hyphothesis
X1	Respondents’ age	1 = Young aged farmers, if age is ≤30years,	+/-
		2 = Old aged farmers, if age is >30 years	
X2	Education	0 = Illiterate (can only sign the name),	+
		1 = Literate (can read, write and sign)	
X3	Household Size	1 = if the household number is ≤4 persons,	+
	(HHS)	2 = if the household number is >4 persons	
X4	Household labor	0 = No, if household do not have extra labor,	-
	(HL)	1 = Yes, if household have extra labor	
X5	Land ownership	1 = if the farmer have full ownership rights,	+
	(LO)	0 = if the farmer do not have ownership rights	
X6	Farm Size	1 = Small, if the farmers land size is ≤ 50 decimal	-
	(FS)	2 = Big, if the farmers land size > 50 decimal	
X7	Topography of Farm	0 = Mid low land,	-
	Land (TFL)	1 = Mid high land	
X8	Secondary Income (SI)	0 = if farmers seasonal secondary income is ≤35000 Taka,	-
		1 = if farmers seasonal secondary income is >35000 Taka	
X9	Knowledge of Recommended Doses	0 = No, if farmers do not know about recommendation doses,	+
	(KRD)	1 = Yes, if farmers know about recommendation doses	
X10	Credit availability (CA)	0 = No, if availing credit is difficult when needed during cropping season,	+
		1 = Yes, if availing credit is not difficult when needed during cropping season	
X11	Soil Water Retention condition (SWR)	0 = Long, if the soil can hold water long,	+
		1 = Not long, if the soil unable to hold water long	
X12	Cow Manure Availability (CMA)	0 = household need to buy cow manure,	+
		1 = household do not need to buy cow manure	
X13	Environmental Awareness (EA)	1 = Yes, if the farmer knows the negative affect of excessive fertilization on soil and environment,	+
		0 = No, Otherwise	
X14	Information Seeking Behavior	0 = No, if farmer do not seeks information of fertilizer application doses from others,	+
	(ISB)	1 = Yes, if farmers seek information of fertilizer application doses from others	

Source: Field Survey Data, 2021

### 2.3 Data analysis

The Chi-square test at a 95 percent confidence level was performed to test the hypothesis through software for statistics and data science (STATA) version 14.0. The test is 2-tailed (non-directional), and in all cases, the null hypothesis (Ho) represents no relationship, while the alternate hypothesis (Ha) assumes a relationship between variables. If the observed *p*-value was less than 0.05, the Ho was rejected and vice-versa. Cramer’s V was measured to determine the strength of relationships. Apart from that, Pearson’s test and likelihood ratio were used to compare the *p*-value to the rejection level when basic chi-square assumptions were violated [73]. We later conducted an ordered probit with the sample selection model that deals with a two-stage decision process regression to determine factors influencing fertilizer adoption and their level of use through employing the ‘heckoprobit’ command in STATA (version 14.1) software. Likewise, the variance-inflation factor (VIF) was estimated to identify the multicollinearity using the ‘vif’ command, and marginal effects were estimated using the ‘margin’ command in STATA. Finally, the results of this analysis have been presented using frequency tables and cross-tabulations.

## 3. Results

### 3.1 Descriptive statistics of the variables

Table 2 illustrates the descriptive statistics of the variables used in the model. The results indicated that out of 405 respondent farmers, 22.22 percent were non-adopters, 39.01 percent were low-bound adopters, 15.8 percent middle-bound, and 22.96 percent high-bound adopters. Significant (p < 0.05) Chi-square test results indicated significant differences between these groups regarding land ownership, farm size, the topography of farmland, credit availability, soil water retention condition, cow manure availability, and information-seeking behavior.

**Table 2:** Descriptive statistics of the explanatory variables.

Variables	Low bound adopters	Middle bound adopters	High bound adopters	Non-adopters (n = 90)	χ2	Cramer’s
	(n = 158)	(n = 64)	(n = 93)		test	V
	*Frequency*	*Frequency*	*Frequency*	*Frequency*		
**Age**					*χ2* = 6.5,	0.126
*Young*	12	8	17	11	*df* = 3,	
*Old*	146	56	76	79	*p* = 0.09	
**Education**					*χ2* = 1.3,	0.057
*Illiterate*	22	7	16	12	*df* = 3,	
*Literate*	136	57	77	78	p = 0.73	
**HHS**					*χ2* = 2.4,	0.077
*≤4 person*	85	37	45	53	*df* = 3,	
*>4 person*	73	27	48	37	*p* = 0.49	
**HHL**					*χ2* = 5.2,	0.114
*No*	138	53	71	76	*df* = 3,	
*Yes*	20	11	22	14	*p* = 0.16	
**LO**					χ2 = 9.9,	0.156
*Not Own*	2	5	6	9	df = 3,	
*Own*	156	59	87	81	p = 0.02*	
**FS**					*χ2* = 13.5,	0.183
*≤50 dec*.	79	30	38	24	*df* = 3,	
*>50 dec*.	79	34	55	66	*p* = 0.00*	
**TFL**					*χ2* = 126.5,	0.559
*Mid Low*	157	60	68	38	*df* = 3,	
*Mid High*	1	4	25	52	*p* = 0.00*	
**SI**					*χ2* = 1.8,	0.066
*≤35000 TK*	55	19	27	33	*df* = 3,	
*>35000 TK*	103	45	66	57	*p* = 0.62	
**KRD**					*χ2* = 2.1,	0.072
*No*	98	42	64	63	*df* = 3,	
*Yes*	60	22	29	27	*p* = 0.56	
**CA**					*χ2* = 11.0,	0.165
*No*	78	27	40	25	*df* = 3,	
*Yes*	80	37	53	65	*p* = 0.01*	
**SWR**					*χ2* = 153.3,	0.615
*Long*	154	51	45	24	*df* = 3,	
*Not Long*	4	13	48	66	*p* = 0.00*	
**CMA**					*χ2* = 17.04,	0.205
*Buy*	18	10	17	29	*df* = 3,	
*Not buy*	140	54	76	61	*p* = 0.00*	
**EA**					*χ2* = 7.5,	0.136
*No*	97	40	60	42	*df* = 3,	
*Yes*	61	24	33	48	*p* = 0.06	
**ISB**					χ2 = 75.0,	0.430
*Yes*	57	54	73	35	*df* = 3,	
*No*	101	10	20	55	*p* = 0.00*	

Source: Field Survey Data, 2021.

Note:

* are statistically significant at 5% (p < 0.05).

The results in Table 2 show that nearly 95 percent of respondents possess full land ownership, while the rest are cultivated on rented land. The farmers that cultivated more extensive farmlands (>50 decimal) constituted 53.12 percent of middle bound adopters, 59.14 percent of high bound adopters, and 73.33 percent of non-adopters. Mid-low land cultivators constitute the highest numbers (90.48 percent), and the water-holding capacity of adopters’ farmland was higher than non-adopters. Regarding availing credit during the cropping season, 49.37 percent of low bound, 42.19 percent of medium bound, 43.01 percent of high bound, and 27.78 percent of non-adopter farmers reported facing difficulties. About 81.73 percent of the respondents also reported not buying cow manure. Most medium-bound adopters (84.38 percent) and high-bound adopters (78.49 percent) sought fertilizer application information from various sources, while most of the non-information-seeking farmers (63.92 percent) were low-bound adopters.

### 3.2. Determinants of adoption

The factors influencing farmers’ initial decision to adopt the BRRI recommended doses and their adoption level were analyzed using the sample selection for ordered probit regression, and the results are presented below (Table 3). As the coefficient result only expresses the direction of change and not the probability or magnitude of change, the marginal effects were thus analyzed (included in Table 3).

**Table 3:** The ordered probit model with sample selection.

Variables	*Selection Model*		*Outcome Model*						VIF
	Adopt or not		Lower Bound		Middle Bound		Higher Bound		
	*dy/dx*	*Std*. *Err*.	*dy/dx*	*Std*. *Err*.	*dy/dx*	*Std*. *Err*.	*dy/dx*	*Std*. *Err*.	
*Age*									1.69
*>30 years*	-0.0113	0.3560	0.0994	0.0751	-0.0084**	0.0041	-0.0910	0.0730	
***Edu***									1.09
*Literate*	-0.0184	0.2871	0.0436	0.0555	-0.0050	0.0055	-0.0386	0.0502	
***HHS***									1.02
*>4 person*	0.0270	0.1641	-	-	-	-	-	-	
***HHL***									1.12
*Yes*	0.0908**	0.2876	-0.0349	0.0525	0.0042	0.0056	0.0307	0.0470	
***LO***									1.09
*Own*	-0.0109	0.3641	0.0628	0.0914	-0.0061	0.0059	-0.0567	0.0860	
***FS***									2.49
*>50 dec*.	0.0062	0.3136	-0.0351	0.0588	0.0047	0.0082	0.0303	0.0507	
***FE***									1.78
*>20 years*	0.0319	0.2521	0.0010	0.0556	-0.0001	0.0074	0.0009	0.0483	
***TNL***									1.80
*Mid high*	-0.2903***	0.2449	-0.3212***	0.0458	-0.0566*	0.0294	0.3778***	0.0719	
***SI***									1.05
*>35000TK*	0.0236	0.1699	-	-	-	-	-	-	
***CA***									2.40
*Yes*	-0.0787	0.3215	-0.0586	0.0576	0.0081	0.0084	0.0505	0.0495	
**SWR**									1.75
*Not long*	-0.2589***	0.2201	-0.3918***	0.0368	-0.0680***	0.0230	0.4597***	0.0531	
***EA***									1.70
*Yes*	-0.1280***	0.2138	-0.0564	0.0493	0.0067	0.0055	0.0497	0.0442	
***KRD***									1.68
*Yes*	0.1134***	0.2314	0.1007**	0.0496	-0.0150*	0.0085	-0.0857**	0.0420	
**CMA**									1.26
*Not buy* *** ***	0.0552	0.2221	0.1296**	0.0517	-0.0095**	0.0047	-0.1202**	0.0517	
**ISB**									1.06
*No*	-0.1914***	0.1963	-	-	-	-	-	-	

Cut 1/Threshold 1: -0.7262* (coefficient), 0.4342 (standard error)

Cut 2/Threshold 2: -0.1102 (coefficient), 0.4335 (standard error)

Number of observations = 405

Censored observations = 90

Uncensored observations = 315

Wald chi2(13) = 146.18

Prob > chi2 = 0.000

Log likelihood = -387.01

LR chi2(1) = 15, Prob > chi2 = 0.000

Note:

*** *p < 0*.*01*

** *p < 0*.*05* and

*p < 0.*10*

The Wald test result statistics (Wald *χ*2 = 146.18 with *p* < 0.000) indicated a good model fit (Table 3). The likelihood-ratio test result *χ*2 (1) = 15 with *p* < 0.000 suggests that we can reject the null hypothesis, and thus, justify the use of the ordered probit sample-selection model over the simple ordered probit model. Moreover, the equality of the cut-points test result assured the rejection of the null hypothesis that the two cut-points were equal. The calculated Variance Inflation Factor (VIF) ranged from 1.02 to 2.49, which was well below the conventional threshold of 10, indicating no issue of multicollinearity [74].

Of the 15 explanatory variables, respondents’ age, household labor availability, type of cultivable lands, soil water retention conditions of farmlands, environmental awareness, knowledge of recommended doses, cow dung purchase condition, and information-seeking behavior have significantly influenced the likelihood of adoption (Table 3). The estimated marginal effect indicates that the chances of being in the lower and middle bound categories decrease by 32.12 percent and 5.66 percent, respectively, for farmers cultivating mid-high land. On the other hand, the probability of farmers’ adopting higher bound fertilizer doses increases by 37.78 percent if they cultivate mid-high land.

The marginal effects result for the soil water retention variable reveals that the likelihood of being in the lower and middle bound category of fertilizer doses decreases by 39.18 and 6.80 percent, respectively, for farmers cultivating in low water holding farmland. Conversely, the probability of being in the higher bound of fertilizer doses category increases by 45.97 percent when farmers cultivate low water holding capacity farmland. Furthermore, the marginal effect value signified that the adoption chances of recommended fertilizer doses increased to 9.08 percent when a household had agricultural labor in the family.

Apart from the aforementioned variables, the likelihood of adoption of recommended doses is significantly higher among farmers who possess knowledge of recommended doses compared to their counterparts who do not. The marginal effect analysis suggested that the likelihood of being in the lower bound category increases by 10.07 percent when a farmer possesses knowledge of recommended doses. The marginal effect of cow dung availability suggested that farmers with adequate inhouse dung supply are 12.96 percent more likely to be in the lower bounds of recommended fertilizer doses. In addition, farmers who are reluctant to seek information on fertilizer application were 19.14 percent less likely to adopt recommended fertilizer doses than their counterparts.

### 3.3 Judgment of soil condition

We were interested in knowing how farmers in our study areas recognized the condition and composition of their soil and whether they had methods to sustain an adequate balance in their soil nutrition. Hence, we thought it would be worth knowing respondents’ positions regarding soil testing since soil analysis provides information about the current nutrient content of the soil and identifies indicators to judge the suitability of specific soils for crop production. Armed with this information, farmers can apply the exact type and quantity of fertilizer needed to improve their soil condition.

The result in Table 4 above shows that even though the majority (96.05 percent) knew about soil-testing facilities, only 1.2 percent had tested their soil in the last five years, and none had been adopted before the current cropping time. Our result indicates that 71.60 percent have gathered knowledge through NGO programs, while the others have acquired knowledge via contract farming organizations (13.33 percent), extension services (6.67 percent), and friends and neighbors (4.44 percent). However, 16 farmers have reported not possessing any knowledge of soil testing facilities. Moreover, the majority (36.30 percent) of respondents’ adoption unwillingness was associated with their thinking of redundancy, reluctancy to carry samples (21.73 percent), dubiety of effectivity (20 percent), the distance of the facility (13.58 percent), cost association (4.44 percent) and unawareness (3.95 percent).

**Table 4:** Respondents’ soil testing status, knowledge and sentiments towards non-adoptions.

*Key factors*	*Non-adopters*	*Lower Bound adopters*	*Middle Bound adopters*	*Higher Bound adopters*
***Knowledge of Soil test***				
*Yes*	*82*	*150*	*64*	*93*
*No*	*8*	*8*	*-*	*-*
***Knowledge Source***				
*NGO*	*59*	*108*	*52*	*71*
*Contract Farming*	*12*	*20*	*5*	*17*
*Extension*	*7*	*16*	*4*	*-*
*Friends or Neighbors*	*4*	*6*	*3*	*5*
*Never heard*	*8*	*8*	*-*	*-*
***Tested Soil in last 5 years***				
*Yes*	*2*	*3*	*-*	*-*
*No*	*-*	*-*	*-*	*-*
***Tested Soil this year before cropping***				
*Yes*	*-*	*-*	*-*	*-*
*No*	*90*	*158*	*64*	*93*
***Reason for not Adopt***				
*Not free*	*5*	*11*	*1*	*1*
*No near facility*	*13*	*20*	*8*	*14*
*Reluctant to carry soil*	*17*	*36*	*19*	*16*
*Did not trust*	*19*	*33*	*13*	*16*
*Not needed*	*28*	*50*	*23*	*46*
*Never heard*	*8*	*8*	*-*	*-*

Source: Field Survey Data, 2021.

We also asked ten experts from the Soil Resources Development Institute (SRDI), two from Grameen Intel, and one from BRRI about the low popularity of soil testing services. The majority (8) of experts thought policymakers’ inadequate focus, lack of lab facilities, and lack of field demonstration activities to earn farmers’ trust were responsible for their lack of engagement in the process. Others believe the irregularity of schemes, unavailability or the high price of soil testing kits, and focusing on a limited number of farmers are responsible for low adoption.

### 3.4 Farmers’ fertilizer application-seeking base

We wanted to understand respondents’ fertilizer application-seeking behavior more deeply since various studies have testified that the knowledge source influences adoption [75,76].

Our inquiry results in Table 5 reveal that most (57.78 percent) farmers apply fertilizer based on their tacit knowledge and previous years’ crop condition, whereas 32.1 percent usually seek fertilizer sellers’ suggestions, 7.65 percent look for friends, family, or neighbors’ suggestions, and 2.47 percent ask extension officers. This result is consistent with the literature that indicates, apart from relying on their own or peer experience, most farmers also seek suggestions from traders on the amount and dose of fertilizer to be applied [77]. However, this result also raises questions about the capabilities and effectiveness of the extension services and indicates the failure of extension to reach the root level.

**Table 5:** The potential bases for the farmers’ decision-making in fertilizer application.

Extension officers	10
Friends, family or neighbor	31
Fertilizer dealers	130
Own idea and previous year condition	234

Source: Field Survey Data, 2021

## 4. Discussion

The descriptive statistics suggested that a significant proportion of the responding farmers had not adopted the recommended doses, which highlighted the need for increased and more appropriate initiatives [6, 22].

Adoption analysis suggested that the availability of family labor relaxes the farmers’ capital constraints and provides support in times of need, hence positively influencing the technology adoption decision [57]. It was also revealed that, compared to upland farmers, comparatively low land farmers are higher adopters of recommended doses of fertilizer [56]. Also, farmers cultivating in comparatively higher lands tend to adopt the higher dosage of fertilizer. The result is meaningful because the Boro rice farming methods require flooded fields [78], and in the case of comparatively high land, holding ponded water is difficult due to leakages and porous conditions of some soils. So, the higher amount of fertilizer usage is linked to a higher rate of fertilizer waste.

The negative association between age and adoption of recommended doses also implied that younger farmers’ more welcoming attitude toward experimentation with new things [41]. This result also implied the importance of further study on factors that influence older farmers’ low adoption decisions in these regions.

Similarly, farmers cultivating low water holding capacity farmlands are less likely to adopt lower and middle-bound fertilizer doses. The result of the analysis urges us to shed light on some facts that might help others understand the situation better. Soil water holding capacity is vital to soil health as soils that can retain a balanced amount of water nourish crops and keep soil organic matter alive. Therefore, soil organic matter (SOM) plays a vital role in increasing water-holding capacity. However, most of the soils of Bangladesh have low organic matter content [79], and to accumulate SOM, the application of fertilizers is needed [80]. As a result, farmers cultivating low-water-holding capacity farmland were more likely to adopt higher fertilizer doses with the belief that it would balance out the deficit.

Farmers’ environmental awareness was negatively linked to the adoption of recommended fertilizer doses. Even though the results contradict our expectations, they draw attention to farmers’ social and psychological risk factors. The majority of smallholding farmers in our study area are subsistence farmers. Their fertilizer application decisions are primarily associated with their higher yield expectations and influenced by the attitudes of neighbors, fertilizer dealers, and friends in their immediate environment [77]. As a result, environmental awareness regarding the practical implementation of recommended doses often fails to overshadow their high yield expectations.

The farmers’ knowledge positively influenced the adoption of recommended doses, which confirms another study’s findings [22]. The results also indicated that the possibility of adopting middle and higher bounds of fertilizer doses decreased when farmers were aware of the recommended doses. Prior studies have suggested that knowledge, ability, attitudes, and motivation differences influence farmers’ behavioral decisions [81]. Such inconsistency might be prevailing among the respondent farmers in our study area, which implied that, although a farmer might possess the knowledge and favorable attitude towards applying fertilizer within the recommended rate, they might not adopt the middle or higher bound of fertilizer doses due to additional factors such as high input cost or unfavorable crop market price.

The use of lower bound fertilizer doses was associated with the availability of manure. This confirmed prior literature that revealed that when both inorganic fertilizer and manure complements are applied, the farm households that have and apply sufficient quantities of manure will not apply higher doses of inorganic fertilizer and vice versa [82]. Adding to this line of inquiry, the negative association between farmers’ information-seeking behavior and adoption implied that a farmer’s reluctance to seek information on fertilizer application led to a lower likelihood of adoption of the recommended level of fertilizer application. Previous research has found that farmers’ information-gaining behavior, knowledge, and attitude toward innovation all influence technology adoption. However, the effectiveness of the adoption process is also dependent on the farmers’ trust in and access to information sources [61, 83].

Farmers’ perception of soil-testing-based facilities’ adoption was unexpected as the finding reveals that farmers’ decision on fertilizer application was based on their assumptions instead of the scientific soil analysis report. The low adoption was due to farmers’ reluctant behavior and the pessimistic attitude of the service providers towards the facility. This result highlights the importance of proper policy support and initiatives to strengthen farmers’ technical know-how and improve extension services to speed up the adoption process.

Farmers’ fertilizer application-seeking behavior analysis revealed that most farmers in our study area, apart from relying on their own or peer experience, also sought fertilizer application advice from traders. Although the results found in this study concurred with another study [74], they raised questions about the capabilities and effectiveness of the extension services whilst highlighting their shortcomings (i.e., education and training skills) that require upgradation in order to reach the grassroots community levels.

## 5. Conclusions and recommendations

This study has attempted to reveal critical factors that influence rice farmers’ adoption or non-adoption decisions of recommended fertilizer doses for BRRI-29 rice production. The study’s findings emphasize the need for specific efforts to convince older and experienced farmers to adopt recommended doses, while technical assistance before cropping may benefit younger and less experienced farmers. However, the acceleration of the adoption process requires establishing a stronger association between recommended fertilizer dose application and the economic and environmental benefit one would gain. Farmers may be encouraged not to over-fertilize if more and more village-level demonstration projects on the benefit of applying recommended doses are carried out. Likewise, farmers need scientific information to adjust their fertilizer use habits in order to improve crop nutrient recovery efficiency. The findings also suggested that concerned authorities should take appropriate steps to expand the use of organic manure in conjunction with chemical fertilizers to reduce unbalanced chemical fertilizer use.

Our respondents’ rare adoption of soil testing-based fertilizer recommendation facilities raises questions about the readiness of service providers; farmers’ knowledge gap of the adoption benefit; and the failure of extension agents. It is worth noting that the site-specific fertilizer application information from online-based technological platforms in Bangladesh is not entirely reliable due to the incorporation of limited land typology segmentation information. Therefore, aggregate land typology and soil water retention information can enhance the efficiency of online fertilizer recommendation technology. It is also essential to understand that without training farmers on how to interpret soil test data and enhance their knowledge of abiotic and biotic factors that affect yield and crop responsiveness to fertilizers, scaling up the adoption of soil testing-based fertilizer application may not occur.

It is evident that the respondents’ trust in extension services is an issue and forces them to rely upon their subjective assumptions and seek advice from other sources when deciding on fertilizer applications. Hence, programs that would improve communication with extension agents and spread knowledge on how fertilizer returns vary by time and quantity are expected to influence their decision on optimum fertilizer use. There may also be a need for assistance from those farm practitioners who follow more scientific fertilizer recommendations in influencing others who are more skeptical about breaking the conventional farming method. This may also build the pool of data for further improvements to be made.

Finally, the role of great marketing initiatives may also bring a huge benefit to the farming community. Promotion of appropriate site and crop-specific customized fertilizer blends, if marketed at competitive prices with proper instruction on usage quantity, may prove to be more effective in reducing farmers’ unbalanced fertilizer use, boosting crop yields, and preventing soil fertility decline in the long run.

This study used only 405 sampled farmers’ information collected from only one district in Bangladesh and could not include data associated with fertilizer purchasing source and application time, micronutrient application methods, and fertilizer availability at peak periods. Therefore, considering these issues as limitations of this study, future research efforts are expected to cover more areas, samples, and variables.

## Supporting information

S1 Data (XLSX)
